# Mis-anaesthetized society: expectancies and recreational use of ketamine in Taiwan

**DOI:** 10.1186/s12889-019-7616-1

**Published:** 2019-10-17

**Authors:** Chao-Ming Chang, Tat Leong Wu, Te-Tien Ting, Chuan-Yu Chen, Lien-Wen Su, Wei J. Chen

**Affiliations:** 1 Institute of Epidemiology and Preventive Medicine, College of Public Health, National Taiwan University, Taipei, Taiwan; 2 Division of Health Technology Assessment, Center for Drug Evaluation, Taipei, Taiwan; 3 School of Big Data Management, Soochow University, Taipei, Taiwan; 4 Department of Public Health, College of Public Health, National Taiwan University, Taipei, Taiwan; 5 Institute of Public Health, National Yang-Ming University, Taipei, Taiwan; 6 Center for Neuropsychiatric Research, National Health Research Institutes, Zhunan, Miaoli County Taiwan; 7 Kunming Prevention and Control Center, Taipei City Hospital, Taipei, Taiwan; 8 Department of Psychiatry, College of Medicine and National Taiwan University Hospital, National Taiwan University, Taipei, Taiwan

**Keywords:** Ketamine, Drug use expectancies, Illicit drugs, Computer-assisted self-interview, Expectancy

## Abstract

**Background:**

The popularity of ketamine for recreational use has been increasing in Asia, including Taiwan. Still, little known about the pattern of ketamine expectancies and whether such patterns are related to ketamine use. This study aimed to examine whether the positive and negative ketamine expectancies are differentially associated with ketamine-using behavior, and whether such relationship may differ by early-onset use of tobacco or alcohol.

**Methods:**

Participants were recruited using respondent-driven sampling (RDS) among regular tobacco and alcohol users, aged 18 to 50, residing in Taipei from 2007 to 2010. Totally 1115 participants (with an age distribution skewed to the right, median = 26; interquartile range: 22–32) had information on substance use and completed a 12-item ketamine expectancies questionnaire (with 6 positive and 6 negative statements). Using two axes of High and Low expectancies, the four combinations of binary positive and binary negative ketamine expectancies were created. Each participant’s drug**-**using experience was categorized into illicit drug naïve, exclusive ketamine use, polydrug ketamine use, or other illicit drug use. Using the weights in the network output by RDS Analysis Tool, multivariable logistic regression analysis was then conducted.

**Results:**

The weighted prevalence was 2.4% for exclusive ketamine use, 9.0% for polydrug ketamine use, and 9.1% for the other illicit drug use. Ketamine users (11.4%) had greater positive expectancies and lower negative expectancies, particularly the combination of High Positive with Low Negative, as compared to the illicit drug-naïve or other illicit drug users. After adjustment for early-onset tobacco (or alcohol) use and sociodemographic characteristics, High Positive, Low Negative, and their combination of High Positive-Low Negative expectancies remained strongly associated with ketamine uses, without evidence of moderation from early-onset use of tobacco or alcohol.

**Conclusions:**

Positive and negative ketamine expectancies were associated in opposite directions with ketamine use, independent of early-onset use of tobacco or alcohol. Our results indicate ketamine expectancies as possible targets for future intervention and prevention of ketamine use, with a less confrontational feedback on decreasing an individual’s positive expectancies is essential in preventing young people from the initiation of ketamine use.

## Introduction

Ketamine, synthesized as a dissociative anesthetic in the 1960s, was initially used for surgical anesthesia during the Vietnam War [1]. Non-medical use of ketamine remained rare until the emergence of “rave” scenes in 1990s, when ketamine was used as an adulterant to ecstasy for recreational use [2]. Since 2000 the popularity of ketamine for recreational use among young people began to increase [3], particularly in Asia [4]. Despite a low dose of ketamine being indicated for treating refractory depression [5], relatively high dose of recreational use of ketamine can lead to a variety of adverse health problems. A well-known psychotomimetic effect of ketamine, so-called “k-hole”, that has made it a popular recreational drug [6] has raised the concern over an increased risk of developing psychotic symptoms persisting far beyond the period of intoxication [7–11]. Repetitive use of ketamine has further led to cognitive impairment [12], increased impulsivity [13, 14], depression [15], ulcerative cystitis [16], and accidental deaths [17]. In addition, ketamine was often used with other drugs simultaneously or in sequence, resulting in even more severe health problems [18]. Some recent studies started to tackle the issue of the intervention for ketamine abuse, such as investigating users’ strategies used to minimize harm from ketamine use [19], developing a severity of dependence scale for ketamine use [20], and demonstrating the efficacy in reducing drug use of a short-term hospitalization and community support program for young people who abused ketamine and were admitted for medical treatment [21].

In Taiwan, the popularity of ketamine surged since early 2000s. Comparing two series of national surveys among school-attending adolescents, the most commonly consumed illicit drugs or inhalants changed from methamphetamine, sniffing glue, and flunitrazepam in the early 1990s [22] to ecstasy, ketamine, and marijuana in the period from 2004 to 2006 [23]. The increasing popularity of ketamine was also found among adolescents surveyed via street outreach [24] and young adults ascertained by respondent driven sampling (RDS) during the period of 2007–2010 [25]. In response to the surge of ketamine use in Taiwan, an amendment in Narcotics Hazard Prevention Act in 2009 stipulated that people who used or possessed ketamine, which is declared as Schedule Three narcotics, of less than 20 g are forced to attend a “narcotics hazard seminar of more than four hours and less than eight hours,” but will be prosecuted for criminal charge if the weight is 20 g or more [4]. Nevertheless, the prevalence of ketamine remained high in 2014 and the characteristics of ketamine users were different from hard drugs users (mainly methamphetamine and heroin), in terms of socio-behavioral correlates and psychosocial distress [26].

Expectancies represent specific anticipated effects from using the substance in question, with positive expectancies reflecting an individual’s attitude toward the outcome as being beneficiary (e.g., relaxation or social disinhibition) and negative expectancies as being harmful (e.g., losing control or getting blamed) [27, 28]. Cumulative efforts have been made to understand the roles of expectancies over the course of substance use disorders [27, 28], and available evidence concerning expectancies with substance use was particularly prolific and comprehensive in alcohol. To this point, positive alcohol expectancies have been consistently linked with a variety of alcohol use behaviors, including increased frequency and quantity of alcohol consumption [28], risky drinking [29], binge drinking, and getting drunk [30], whereas the effects of negative alcohol expectancies are rather mixed [31–33]. Furthermore, several factors that might shape a person’s endorsement of alcohol expectancies, particularly in young population, have been examined. Positive alcohol expectancies were found to increase and negative alcohol expectancies generally decline during adolescence when the risk of alcohol initiation and problematic drinking escalates [34–36]. Other than developmental stage or age, predictors of alcohol expectancies included an array of individual characteristics (e.g., gender, pubertal development, genetics) and environmental factors (e.g., parental drinking, peer drinking, peer network, and alcohol advertisement) [35–41].

Expectancies for a particular substance might be established indirectly (e.g., mass media, social norm, or seeing perceived drug effects from others) for those who did not use the substance [42]. Therefore, expectancies could be a predictor for the substance use among people who have not yet experienced the substance. In addition, once people begin to use certain substance, its direct effects might influence the existing expectancies [35]. Thus, a change in expectancy might affect an individual’s decision to keep using the substance or trying to get sober, and could serve as the means for alleviating people’s use of substance [42–44].

Drug expectancies have been conducted on marijuana and cocaine use [42, 45–48]. Otherwise, research of expectancies on illicit drugs is sparse [49]. To date, only one study has examined negative expectancy of ketamine using a single item among Taiwanese adolescents [50]. It remains little known about the pattern of expectancies toward ketamine use and whether such expectancies are related to ketamine use.

A majority of illicit drug users have used cigarettes [51] or alcohol [52] prior to the initiation of any illicit drugs. Prospectively speaking, early-onset use of tobacco [53, 54] or alcohol [55–57] was associated with further involvement in illicit drug use. The so-called gateway substances, i.e., legal substances (e.g., alcohol and tobacco) serving as gateway drugs for illicit drug use [58–61], have been implicated to affect one’s further illicit drug involvement through cognitive, neurological, and social processes. Nevertheless, it remains unknown whether recreational use of ketamine was associated with early-onset use of tobacco or alcohol, and whether the relationship between ketamine expectancies and ketamine use was modified by such early-onset use.

To fill in the gaps in the literature, this study examined the ketamine expectancies among adults in Taipei with different levels of ketamine use, i.e., illicit drug-naive, exclusive ketamine use, polydrug ketamine use, and the other illicit drugs use. The specific aims of this study were to evaluate: 1) whether the positive and negative ketamine expectancies were differentially associated with ketamine-using behavior, 2) whether early initiation of tobacco or alcohol use was associated with increased risk of ketamine use, and 3) whether early initiation of tobacco or alcohol use moderated the relationship between ketamine expectancies and ketamine use.

## Methods

### Study sample

Participants of this study were recruited using respondent driven sampling (RDS) among alcohol- and tobacco-using adults in Taipei metropolitan area from 2007 to 2010, with more detail about the design, participants, and measurements being described elsewhere [25]. Briefly, the initial seeds were either community-based (35 nightclub customers or KTV attendees) or hospital-based (12 patients enrolling in drug rehabilitation), and every seed was asked to recruit their friends who were (a) adult residents living in the Taipei metropolitan area with an age preferably less than 50; and (b) regular alcohol and tobacco users. Despite the variation in seed sources, subsequent respondents were recruited primarily on the basis of their substance-using network, without any confinement to the initial setting. Neither the initial seeds nor subsequent recruiters were asked to identify illicit drug users in the referral procedure to make potential recruits less wary of potential identification regarding their illegal drug use. Nevertheless, most of the sample proportions of illegal drug use converged to equilibrium proportions around the third wave in our RDS sample, in support of our implementation of RDS in epidemiological studies on illegal drug use [25].

Participants were informed the nature of the study and were guaranteed confidentiality prior to the survey. Afterwards, written informed consent was obtained from all participants. Participants were offered NTD 300 (around USD 10) worth of convenience store vouchers upon completion of the interview and were further offered NTD 100 worth of vouchers for every peer they successfully recruited into the study. To balance the need for referral and confidentiality, each participant was asked about his or her nickname and preferred way of communication (mostly mobile phone number). The relationship of new recruits to the network was then verified by inquiring about the nickname of their recruiter. For every recruit, we adopted a research identification numbering system to denote the social relationship and the order in the referral chains. By doing this, we could collect the information to establish the social network and prevent the same person from entering the sampling more than one time. Then the recruitment phase was repeated until equilibrium, i.e., an estimate derived from the transition probability at equilibrium, was attained (about another eight waves). The study was approved by the institutional review board of the College of Public Health, National Taiwan University.

Due to budget constraints, instead of carrying out the RDS in a single implementation this study was conducted on a yearly basis by using the same guidelines each year. The numbers of participants recruited in this fashion was 144 in 2007, 328 in 2008, 350 in 2009, and 293 in 2010. Before aggregating samples from different years together, we examined the distribution of lifetime ketamine use and male gender in different years (Additional file 1: Table S1). The results revealed that except the first year, which was the preparation year and had the smallest number of recruits, the prevalence of ketamine and male gender were quite similar across years. Thus, we pooled the samples from different years together, with an aggregated sample size of 1115 persons. As reported previously, the RDS-adjusted estimates for ever using ketamine in the aggregated sample already reached equilibrium at wave three [25].

### Measurement

Each participant underwent an audio computer-assisted self-interview (ACASI) implemented on notebook computers. The questionnaire consists of four sections: (1) social demographic characteristics; (2) risk-taking behaviors, including use of licit substances and illicit drugs/inhalants, and risky sexual experience; and (3) expectancies recognized as the effects of drug use.

#### Early onset of tobacco or alcohol use

Since the compulsory education system in Taiwan covers the first 9 years of education, i.e., middle or junior high school, students typically attend schools in neighborhood. Thus, initiation of tobacco or alcohol use before age 16, i.e., before attending senior high school, are considered as early onset, which was younger than the legal age (18 years old) of purchasing tobacco or alcohol in Taiwan.

#### Drug use history

The measures on illicit drugs/inhalants included 9 categories (i.e., ketamine, ecstasy, super glue, methamphetamine, flunitrazepam [so-called FM2], marijuana, heroin or morphine, angel dust, gamma hydroxybutyrate [GHB]). For respondents with any use of illicit drug experience, further inquiries were made regarding age at first use, the first use setting, use frequency, social function impairment, and treatment-seeking behaviors [23]. Each participant’s drug-using experience was categorized into four groups: 1) illicit drug naïve, 2) exclusive ketamine use, 3) polydrug ketamine use, and 4) other illicit drug use.

#### Ketamine expectancy

Since there has been no ketamine expectancy questionnaire, we decided to develop one by modifying cannabis expectancy questionnaire for the following considerations: (1) ketamine often plays the role of the first illicit drug in Taiwan since early 2000s [23–25], similar to that of cannabis in western society. In Taiwan, ketamine was commonly seized in recreational settings (e.g., night clubs) with relaxation, joy, and out of reality touch being the mostly mentioned drug effects. For those who never used ketamine, their expectancies toward ketamine were mostly acquired from media portray and social network [62]; (2) drug expectancies have long been demonstrated to converge upon a common construct, with positive expectancies being a more powerful motivator for substance use than negative expectancies [63].

Thus, we followed the approach of Willner [64] in treating ketamine as an illicit drug after the gateway substance of alcohol, in which he constructed a parallel 26-item Adolescent Cannabis Expectancies Questionnaires using the items as the Adolescent Alcohol Expectancies Questionnaire except replacing “drinking” or “drinking alcohol” by “cannabis,” “smoking cannabis” or “smoking a joint.” After principal component analysis, Willner [64] retained six items for each subscale of Positive Expectancies and Negative Expectancies, with four of the positive items and five of the negative items being common to alcohol and cannabis. Hence, we constructed a ketamine expectancy questionnaire based on the 12-item Adolescent Cannabis Expectancy Questionnaires [64] by replacing cannabis with ketamine. Briefly, these true/false items consist of: (1) six positive expectancies, i.e., having ketamine is a nice way to enjoy a holiday, lets you join in with others who are having fun, helps you stand up to others, makes the world a better place, makes parties more fun, drive better after a joint; and (2) six negative expectancies, i.e., lose control and have accidents, don’t understand things, have trouble remembering, break and destroy things, tend to have a go at kids who are using ketamine (i.e., getting blamed for using ketamine), and makes people less friendly. The internal consistencies (Cronbach’s α) of the ketamine expectancy questionnaire in this study, 0.73 for the positive expectancy and 0.87 for the negative expectancy, were equivalent to those reported in the original cannabis questionnaire [64]. In the confirmatory factor analysis of a two-factor model, the adjusted goodness of fit index was 0.90, and the root mean square error of approximation was 0.08, and comparative fit index was 0.92, indicating acceptable fits. Part of the results of ketamine expectancies was summarized in a 2013 conference [65].

To assess whether some items of ketamine expectancies were more differential in their association with ketamine use, we conducted a series of univariate logistic regression analysis of any ketamine use (versus illicit drug-naïve) for individual items, with an odds ratio (OR) being significant for four positive expectancies and four negative expectancies (Additional file 1: Table S2). Comparing the area under ROC curve in different models, the value was 0.674 for a model of three positive expectancies and 0.776 for a model of four positive and four negative expectancies using backward selection (Additional file 1: Table S3). Hence, the contribution of individual expectancies was incremental and all the items were used in the subsequent analyses.

Since the distributions of the ketamine expectancies were highly skewed, it was difficult to interpret the meaning of a numeric point in expectancies. Hence, we chose the median of the illicit drug-naïve as the cut-off to evaluate whether an individual’s positive or negative expectancies was relatively high or low, i.e., a binary High (≥ median) or Low (< median) expectancies. With differential endorsements between positive and negative expectancies, the median used to divide a sum expectancy into High versus Low expectancies was quite different (1 for the positive expectancies and 5 for the negative expectancies). In other words, an individual endorsing any item out of 6 positive expectancies would be classified as having High Positive expectancies, whereas an individual endorsing all or 5 out of 6 negative expectancies would be classified as having High Negative expectancies. For comparison, we also run the model that included ketamine expectancies as continuous.

### Data analysis

#### RDS-weighted analyses

We computed RDS-weighted prevalence estimates using RDS Analysis Tool (RDSAT) version 7.1 [66]. The 95% confidence intervals (CIs) were estimated with 15,000 bootstrap resamples following the recommendation of the software. Other estimation options remained default. Furthermore, sampling weights reflecting the recruitment patterns of this RDS were incorporated in subsequent analyses. We adopted one recommendation to apply sampling weight of the dependent variable (i.e., ketamine use) to the regression model concerned [67].

#### Group comparisons

For categorical variables, χ^2^ tests or Fisher’s exact tests were used for group comparisons, and a Tukey-type multiple comparison for proportions [68] or multiple comparisons with Fisher’s combination test were used for the relevant post hoc analyses. For continuous variables, t-tests were used in 2 group comparisons, and ANOVA/ANCOVA were used in 3 group comparisons, with Tukey’s HSD tests and Tukey-Kramer adjustments for the post hoc pairwise comparisons.

#### Weighted logistic regression analysis

Using the weights in the RDS network output by RDSAT, we built multivariable logistic models to regress ketamine use on ketamine expectancies, sociodemographic variables, and other substance use experience, as well as potential interaction between early-onset tobacco (or alcohol) use and ketamine expectancies, with adjusted OR (aOR) and its 95% CI being reported.

All statistical tests were two-sided, and a *p*-value < 0.05 was considered significant. All of statistical analyses were performed using SAS 9.2 (SAS Institute, Cary, NC).

## Results

As displayed in Table 1, the study sample had a slightly higher proportion (57.2%) of males, 67.3% having an educational level of less than college, and only 8.7% being unemployed. Among them, 32.0% had early-onset use of tobacco and 34.1% early-onset use of alcohol. The distribution of age was skewed to the right, with the mean (27.9; SD = 7.9) greater than the median (26; interquartile range: 22–32). Meanwhile, the mean age at first use was 16.7 for tobacco and 15.7 for alcohol, with the median age at first use being 16 for both tobacco and alcohol.

**Table 1:** Demographic characteristics and ketamine-using history of the RDS-sample in Taipei Metropolitan Area (*N* = 1115)

Variable	N^c^	%_wt_^d^	95% CI
Gender			
Male	689	57.2	(51.5–62.9)
Female	426	42.8	(37.1–48.5)
Education			
< college	701	67.3	(62.7–72.8)
College and above	414	32.1	(26.8–37.3)
Employment			
Full-time job	631	54.8	(48.6–60.5)
Work-study/in school	345	29.9	(24.5–35.6)
Part-time job/military	60	6.5	(4.5–8.6)
Unemployed	79	8.7	(5.9–12.6)
Early-onset^a^ tobacco use	406	32.0	(27.7–36.4)
Early-onset^a^ alcohol use^b^	427	34.1	(30.1–38.5)
Age in years			
Mean (SD)	1115	27.9	7.9
Median (25–75%)	1115	26.0	22.0–32.0
Age at first tobacco use			
Mean (SD)	1040	16.7	3.6
Median (25–75%)	1040	16.0	14.0–18.0
Age at first alcohol use			
Mean (SD)	1037	15.7	4.1
Median (25–75%)	1037	16.0	14.0–18.0

^a^An onset use age of < 16 years, which is the end of compulsory education up to middle school, is defined as early–onset use.

^b^Three individuals were missing on the information on onset age of alcohol use.

^c^Seeds are included in the numbers.

^d^Weighted percentages and their 95% confidence intervals are RDS-adjusted population proportions estimated using the software RDSAT version 7.1.

### Groups by ketamine-using experience

**Fig. 1:**
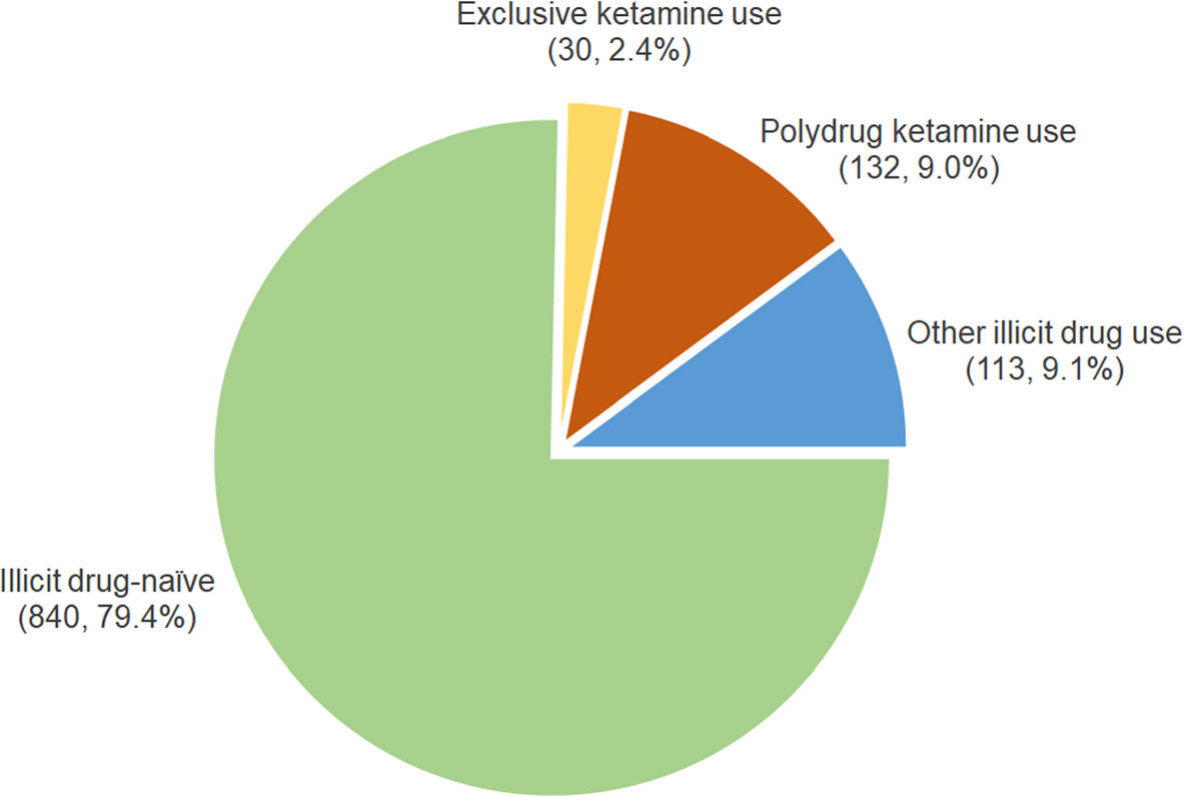
The distribution of different groups of ketamine-using experience among the RDS-sample in Taipei Metropolitan Area (*N* = 1115)

Among the 1115 participants, their ketamine-using experience is depicted in Fig. 1, with 2.4% being exclusive ketamine users, 9.0% polydrug ketamine users, and 9.1% the other illicit drug users. Among the concurrently used illicit drugs for the polydrug ketamine users, the most common one was ecstasy (*n* = 112, 84.6%), followed by marijuana (*n* = 90, 72.4%), methamphetamine (*n* = 39, 26.9%), flunitrazepam (*n* = 18, 11.3%), and heroin (*n* = 14, 4.6%).

Compared to the group of illicit drug-naïve, both polydrug ketamine users and other illicit drug users had a higher proportion of unemployment, early-onset tobacco use, and early-onset alcohol use, and exclusive ketamine users had a higher proportion of early-onset tobacco use (Table 2). Furthermore, the three groups of illicit drug users, as compared to the group of illicit drug naïve, had earlier initiation age of tobacco use, and polydrug ketamine users further had earlier initiation age of alcohol use. There were no significant differences among the four groups in gender and educational level.

**Table 2:** Demographic characteristics and ketamine-using history of the RDS-sample in Taipei Metropolitan Area (*N* = 1115), by illicit drug use experience

Variables	Illicit drug-naïve (Group 1; *N* = 840)			Exclusive ketamine use (Group 2; *N* = 30)			Polydrug ketamine use (Group 3; *N* = 132)			The other illicit drug use (Group 4; *N* = 113)			Group comparisons	
	N	%_wt_	95% CI	N	%_wt_	95% CI	N	%_wt_	95% CI	N	%_wt_	95% CI	P^c^	Post-hoc^d^
Male	504	55.4	(49.3–62.0)	10	63.2	(37.6–86.4)	44	59.7	(45.9–73.8)	36	64.5	(50.2–79.6)	.160	–
Education < college	513	65.9	(60.0–71.5)	23	83.0	(63.2–96.8)	94	71.3	(57.6–82.5)	71	69.6	(55.6–81.6)	.141	–
Unemployment	39	5.8	(3.4–8.4)	3	10.8	(0.0–29.0)	16	14.5	(3.7–28.5)	21	22.6	(7.8–39.6)	< .001	3, 4 > 1
Early-onset^a^ tobacco use	265	26.7	(22.0–30.9)	17	61.8	(38.2–85.9)	72	47.5	(35.7–62.1)	52	52.5	(37.1–63.8)	<.001	2, 3, 4 > 1
Early-onset^a^ alcohol use^b^	290	29.8	(25.4–34.9)	15	47.9	(23.7–74.4)	64	45.8	(33.5–58.8)	58	57.6	(43.4–70.0)	<.001	3, 4 > 1
	N	Mean	SD	N	Mean	SD	N	Mean	SD	N	Mean	SD	P^c^	Post-hoc^d^
Age in years	840	28.1	8.2	30	21.8	3.6	132	26.0	5.5	113	30.1	7.6	< .001	1 > 2, 3; 4 > 3 > 2
Age at first tobacco use	772	17.1	3.7	29	14.6	2.1	129	15.4	3.3	110	15.6	3.0	< .001	1 > 2, 3, 4
Age at first alcohol use	766	16.0	4.1	29	14.9	3.1	130	14.6	3.5	109	15.2	3.9	< .001	1 > 3

^a^An onset use age of < 16 years, which is the end of compulsory education up to middle school, is defined as early–onset use

^b^Three individuals were missing on the information on onset age of alcohol use

^c^χ^2^ test or Fisher’s exact test for categorical variables; ANOVA for quantitative variables

^d^Tukey’s HSD test in ANOVA; a Tukey-type multiple comparison for proportions in a 2*4 cross-tabulation for categorical variables (Elliott and Reisch 2006)

Both groups of ketamine users were comparable in the experience of recent use, frequency of use, or age at first use except that polydrug ketamine users had higher proportion of having a lifetime use ≥5 times than the exclusive ketamine users (Additional file 1: Table S4).

### Ketamine expectancies

The proportions of endorsement from participants were lower for positive expectancies than for negative expectancies (Table 3). The sum of positive expectancies was lowest for the illicit drug-naïve, then increased for the three illicit drug-using groups. Meanwhile, the sum of negative expectancies was highest for the illicit drug-naïve, then decreased for the three illicit drug-using groups. When the median of the illicit drug-naïve (1 for the positive expectancies and 5 for the negative expectancies) was used as the cut-off point to define binary High (≥ median) or Low (< median) expectancies, the polydrug ketamine users had the highest proportion of High Positive expectancies, and all of three illicit drug-using groups had lower proportion of High Negative expectancies than the illicit drug-naive did.

**Table 3:** Positive and negative ketamine expectancies of the RDS-sample in Taipei Metropolitan Area (*N* = 1115), by illicit drug use experience

Ketamine expectancies	Illicit drug-naïve (Group 1; *N* = 840)		Exclusive ketamine use (Group 2; *N* = 30)		Polydrug ketamine use (Group 3; *N* = 132)		The other illicit drug use (Group 4; *N* = 113)		Group comparison	
	%_wt_	95% CI	%_wt_	95% CI	%_wt_	95% CI	%_wt_	95% CI	P	Post-hoc^b^
P1 (stand up to others)	11.2	(7.9–14.8)	22.5	(7.7–44.0)	25.5	(16.0–39.9)	12.0	(4.6–21.2)	<.001	3 > 1, 4
P2 (join in with others)	46.0	(40.7–51.5)	63.7	(38.9–83.4)	76.1	(66.6–86.1)	41.6	(26.8–53.3)	<.001	3 > 1, 4
P3 (drive better)	4.7	(2.4–7.6)	19.2	(1.4–41.5)	1.0	(0.0–2.7)	7.6	(0.0–17.2)	<.001	2 > 1, 3; 4 > 3
P4 (make parties more fun)	35.5	(30.9–40.6)	64.5	(37.8–83.6)	72.7	(64.5–84.0)	51.8	(38.0–65.0)	<.001	3 > 1, 4; 2, 4 > 1
P5 (enjoy a holiday)	20.7	(16.7–25.2)	31.9	(7.7–54.2)	27.9	(19.1–40.9)	26.3	(13.1–37.5)	.074	
P6 (make the world a better place)	13.7	(10.4–17.5)	15.4	(1.9–33.6)	19.3	(10.3–30.5)	22.6	(9.6–37.6)	.041	
N1 (lose controls and have accidents)	71.2	(66.3–76.5)	51.2	(29.2–79.2)	54.8	(40.8–65.4)	56.0	(40.9–69.3)	<.001	1 > 3, 4
N2 (make people less friendly)	51.4	(46.2–57.1)	28.4	(12.4–53.2)	18.6	(9.8–27.7)	39.5	(25.2–51.6)	<.001	1, 4 > 3
N3 (have a go at kids who are using)	69.5	(64.2–75.3)	58.6	(34.9–82.0)	74.4	(63.0–84.4)	61.3	(43.8–73.5)	.099	
N4 (don’t understand things when using)	66.8	(60.9–72.5)	73.0	(52.0–90.4)	61.8	(48.9–73.1)	52.4	(36.5–64.4)	.014	1 > 4
N5 (break and destroy things when using)	67.9	(62.6–73.6)	35.9	(17.7–63.1)	41.3	(28.1–55.2)	49.0	(33.6–60.2)	<.001	1 > 2, 3, 4
N6 (have trouble remembering)	74.3	(69.3–79.9)	77.4	(57.5–94.4)	74.4	(63.8–83.6)	64.5	(48.3–77.3)	.170	
Summary of expectancies										
Positive sum, mean (SD)	1.4	(1.5)	2.1	(1.6)	2.3	(1.5)	1.7	(1.6)	<.001	3 > 1
Negative sum, mean (SD)	4.2	(2.2)	3.5	(2.2)	3.1	(1.7)	3.6	(2.3)	<.001	1 > 2, 3; 4 > 2 > 3
High Positive (≥ 1)^a^, n (%)	53.5	(48.3–58.9)	71.8	(47.5–89.6)	86.0	(79.3–94.1)	70.3	(56.4–80.3)	<.001	3 > 1,4; 4 > 1
High Negative (≥ 5)^a^, n (%)	59.1	(53.5–64.9)	28.5	(12.6–55.0)	30.3	(16.9–43.4)	46.4	(31.5–58.6)	<.001	1 > 2, 3, 4
Combination of expectancies, n (%)									<.001	
Low Positive-High Negative	17.3	(14.1–21.6)	9.2	(0.0–25.3)	0.8	(0.1–2.0)	12.3	(4.8–21.0)		
Low Positive-Low Negative	29.3	(23.4–34.9)	19.8	(2.9–39.6)	12.8	(5.5–20.5)	17.5	(10.0–29.5)		
High Positive-High Negative	41.5	(36.1–46.6)	19.2	(6.4–42.5)	29.8	(16.1–42.6)	34.9	(21.3–45.8)		
High Positive-Low Negative	11.9	(8.9–15.4)	51.8	(22.3–72.4)	56.6	(44.4–71.3)	35.3	(20.9–51.1)		

^a^The median of the illicit drug-naïve as the cut-off, with High Positive being ≥ the median (i.e., 1) and High Negative being ≥ the median (i.e., 5)

^b^Tukey’s HSD test in ANOVA; a Tukey-type multiple comparison for proportions in a 2*4 cross-tabulation for categorical variables (Elliott and Reisch 2006)

**Fig. 2:**
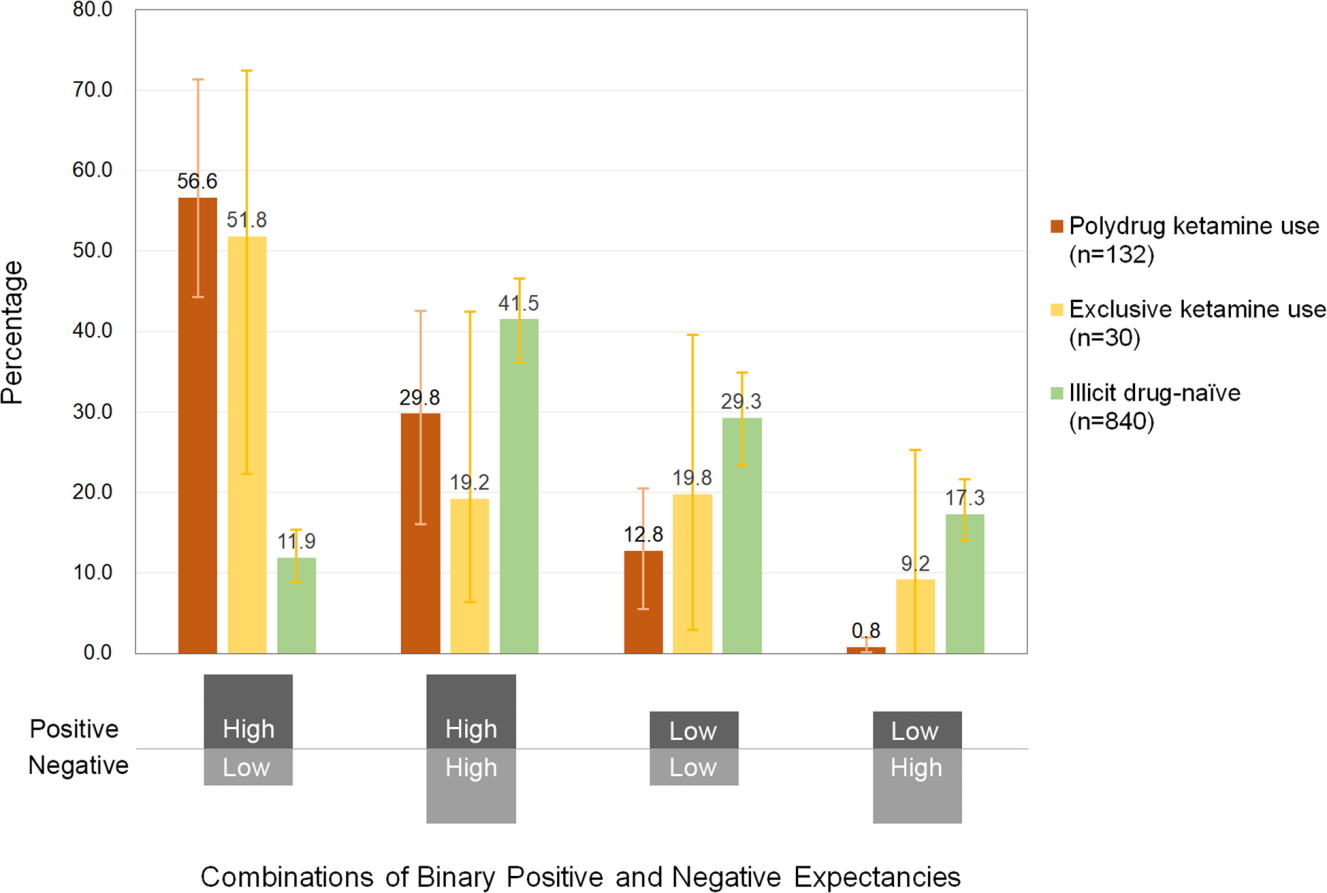
The distribution of the four combinations of binary positive and negative ketamine expectancies, i.e., High-Positive/Low-Negative (HpLn), High-Positive/High-Negative (HpHn), Low-Positive/Low-Negative (LpLn), and Low-Positive/High-Negative (LpHn), among the three groups of different ketamine-using experiences. The cutoff-points were the median of positive expectancies and negative expectancies, respectively, among those who were illicit drug-naïve (i.e., High-Positive as ≥1, High-Negative as ≥5). The vertical bar indicates the 95% confidence interval derived from RDS-weighted estimates

Table 3 also displays the distributions of four combinations of binary positive and binary negative ketamine expectancies. To highlight the contrast, Fig. 2 displays the proportions of individual combinations of binary positive and negative expectancies. The proportion of having High-Positive/Low-Negative expectancies was highest among polydrug ketamine users (56.2%), followed by exclusive ketamine users (51.8%), and then the illegal drug naïve (11.9%). In contrast, the proportions of having Low-Positive/High-Negative expectancies across the three groups were in opposite direction, i.e., lowest among polydrug ketamine users (0.8%), followed by exclusive ketamine users (9.2%), and then the illegal drug-naïve (17.3%).

### Early-onset tobacco or alcohol use and ketamine expectancies

Compared to late-onset tobacco users, early-onset tobacco users had greater positive sum expectancies and lower negative sum expectancies of ketamine. Otherwise, both groups were not different in the distribution of binary positive, binary negative expectancies, and the four combinations of positive and negative expectancies (Additional file 1: Table S5). Meanwhile, compared to late-onset alcohol users, early-onset alcohol users had a borderline decrease in negative sum expectancies, a lower proportion of High Negative expectancies, as well as higher proportions of Low Positive-Low Negative and High Positive-Low Negative expectancies.

### Relations of ketamine expectancies to ketamine use

For the multinomial logistic regression analysis, we combined the group of exclusive ketamine use, which had a small sample size, with polydrug ketamine use to form a pooled group of any ketamine use. Owing to exploratory nature, ketamine expectancies were examined either in binary forms (High Positive and High Negative) or four-combinations, but not in continuous scale. To avoid collinearity, either early-onset tobacco use or early-onset alcohol use was added as a covariate since the two had a high tetrachoric correlation of 0.647 (*p* < 0.001).

Table 4 displays the results of the multivariable multinomial logistic regression of ketamine-using experience (illicit drug-naïve, any ketamine use, and other illicit drug use) on ketamine expectancies in two models, in which expectancies were treated as two binary variables (Model 1), or as a 4-level categorical variable (Model 2). Both models were adjusted for early-onset tobacco use and sociodemographic covariates, including male gender, an educational level of < college, unemployment, and age. In Model 1, people with binary High Positive expectancies had an increased risk of illicit drug use, with aOR being 10.04 for any ketamine use and 3.10 for other illicit drug use. Whereas people with binary High Negative expectancies had a decreased risk of illicit drug use, with aOR being 0.15 for any ketamine use and 0.40 for other illicit drug use. In contrast, the magnitudes of aOR of unemployment and early-onset tobacco use with any ketamine use (4.76 and 2.39) were very similar to those with other illicit drug use (5.43 and 2.44). In Model 2, compared to people with the combination of Low Positive-High Negative ketamine expectancies, people with High Positive-Low Negative expectancies had much increased aORs, followed by those with High Positive-High Negative (5.40) and those with Low Positive-Low Negative (3.85). In contrast, only people with High Positive-Low Negative expectancies had an increased aOR with other illicit drug use of smaller magnitude as compared that with any ketamine use. The magnitude of association for unemployment and early-onset tobacco use with the other illicit drug use remains similar to those of Model 1.

**Table 4:** Multinomial logistic regression model of illicit drug use experience (reference group: illicit drug-naïve) on binary ketamine expectancies with adjustment for sociodemographics and early-onset tobacco use (*N* = 1115)

Variables	Any ketamine use		The other illicit drug use	
	aOR	95% CI	aOR	95% CI
*Model 1*				
Male	1.20	(0.68–2.12)	1.27	(0.71–2.28)
Education < college	1.48	(0.83–2.61)	0.82	(0.45–1.49)
Unemployment	**4.76**	**(1.59–14.19)**	**5.43**	**(2.54–11.6)**
Age in years	**0.94**	**(0.91–0.98)**	1.01	(0.98–1.04)
Early-onset tobacco use	**2.39**	**(1.36–4.21)**	**2.44**	**(1.39–4.28)**
High Positive expectancies	**10.04**	**(5.23–19.27)**	**3.10**	**(1.55–6.22)**
High Negative expectancies	**0.15**	**(0.08–0.26)**	**0.40**	**(0.21–0.76)**
*Model 2*				
Male	1.20	(0.67–2.14)	1.26	(0.7–2.26)
Education < college	1.46	(0.82–2.61)	0.81	(0.45–1.48)
Unemployment	**4.70**	**(1.60–13.76)**	**5.25**	**(2.52–10.93)**
Age in years	**0.94**	**(0.91–0.98)**	1.01	(0.98–1.04)
Early-onset tobacco use	**2.45**	**(1.37–4.36)**	**2.53**	**(1.45–4.39)**
Ketamine expectancy combination (ref: Low Positive-High Negative)				
Low Positive-Low Negative	**3.58**	**(1.13–11.35)**	0.87	(0.36–2.07)
High Positive-High Negative	**5.49**	**(1.75–17.23)**	1.24	(0.56–2.74)
High Positive-Low Negative	**43.47**	**(14.55–129.86)**	**4.87**	**(1.96–12.14)**

Note: (1) high or low expectancies are divided by the median of the illicit drug-naïve

(2): statistically significant results are highlighted in bold

The trends of binary ketamine expectancies with either ketamine use or the other illicit drug use, i.e., increased risk for High Positive and lowered risk for High Negative, were replicated in the models that treated expectancies as continuous (Model 1 in Additional file 1: Table S6). Under this circumstance, however, it was difficult to interpret the interaction term because of the opposite directions of positive and negative expectancies on ketamine use (Model 2 in Additional file 1: Table S6).

When the covariate was changed to early-onset alcohol use, the results of the multivariable multinomial logistic regression analyses were very similar to Table 3 except that the aOR of early-onset alcohol use failed to reach statistical significance for any ketamine use in both Model 1 and Model 2 (Additional file 1: Table S7).

When the interaction terms between early-onset tobacco (EOT) use and binary ketamine expectancies, i.e., High Positive expectancies (HPE) and High Negative expectancies (HNE), were added to Model 1, the two interaction terms, EOT x HPE and EOT x HNE, did not reach statistical significance (Additional file 1: Table S8). Similarly, when the interaction terms between EOT use and ketamine expectancy combinations, i.e., Low Positive-Low Negative (C1), High Positive-High Negative (C2), and High Positive-Low Negative (C3), were added in Model 2, the three interaction terms, EOT x C1, EOT x C2, and EOT x C2, did not reach statistical significance.

Similarly, the interaction terms involving early-onset alcohol use turned out to be non-significant too, and the results are displayed in Additional file 1: Table S9.

## Discussion

This study aimed to examine whether the positive and negative ketamine expectancies are differentially associated with ketamine-using behavior, and whether such relationship may differ by early-onset use of tobacco or alcohol. We found that ketamine users had greater positive expectancies and lower negative expectancies, particularly the combination of High Positive with Low Negative, as compared to the illicit drug-naïve or the other illicit drug users. High Positive expectancies, Low Negative expectancies, and their combination of High Positive-Low Negative expectancies were strongly associated with ketamine use. Further examination of the interaction between early-onset tobacco (or alcohol) use and ketamine expectancies did not detect any synergistic effect between them on ketamine use. These findings provide support for the association of ketamine expectancies with ketamine use independent from other correlates, indicating possible cognitive targets for future intervention and prevention.

In this RDS sample of tobacco- and alcohol -using adults in the Taipei metropolitan area, the weighted prevalence of their illicit drug use was much higher than the estimate of 1.29% in the general population [26]. Majority of our participants had already been aware of more than half of the negative expectancies, i.e., with a mean of negative sum expectancies of > 3.0 even for ketamine users. In contrast, only individuals who had ever used ketamine would endorse more than two positive expectancy items. Furthermore, the mean of positive sum expectancies and negative sum expectancies for the group of other illicit drug use fell in-between that of the group of illicit drug-naïve and the two groups of ketamine use, indicating the expectancies were to some extent specific to ketamine use.

Among potential confounders that were controlled for in our multinomial logistic regression analysis, unemployment and early-onset use of tobacco had significant associations with both ketamine use and other illicit drug use. Nevertheless, the magnitude of aORs of these correlates were similar between ketamine use and other illicit drug use, meaning that they were non-specific risk factors for any illicit drug use. In contrast, the magnitude of aORs of positive or negative ketamine expectancies were much greater for ketamine use than for the other illicit drug use. Under these circumstances, the exploratory questionnaire of ketamine expectancies used in this study did have discriminatory validity.

Our findings about the increasing trend in positive sum expectancies from the illicit-drug naïve to exclusive ketamine use, then to polydrug ketamine use as well as the increased aORs of ketamine use for positive sum expectancies in the multinomial logistic regression analysis are consistent with previous studies showing that positive expectancies might increase people’s substance use or progress to more severe use once they begin to use the drug [30, 45, 48, 69]. However, another possibility is that prior use of ketamine might influence individuals’ expectancies toward the use of ketamine, as indicated in the effect of the initiation of alcohol use on subsequent changes in alcohol expectancies in longitudinal studies [35]. Owing to the cross-sectional nature, this study could not disentangle whether the increased positive expectancies of ketamine users pre-existed before their initiation of ketamine use or enhanced after their use of ketamine. Furthermore, negative expectancies might be inversely associated with ketamine use, though the magnitude for negative expectancies (at least endorsing 5 out of 6 negative expectancies) to exhibit the inverse association was greater than that for its counterparts in positive expectancies (endorsing any 1 out of 6 positive expectancies) to exhibit the positive association. Another feature of this study is that a joint influence of positive and negative expectancies on ketamine use were evaluated. In particular, the combination of High Positive expectancies and Low Negative expectancies poses the greatest risk of any ketamine use. Meanwhile, the combination of Low Positive-High Negative expectancies represents the least risk for ketamine use.

Our findings also revealed that early-onset tobacco use as well as early-onset alcohol use were indeed associated with illicit drug use, regardless of ketamine or other illicit drugs. Both forms of tobacco and alcohol early-onset use were also associated with greater positive expectancies and lower negative expectancies of ketamine. Nevertheless, our multivariable logistic regression models found that early-onset tobacco use (or early-onset alcohol use) and ketamine expectancies had independent associations with ketamine use, and early-onset tobacco use (or early-onset alcohol use) did not modify the association of ketamine expectancies with ketamine use. Hence, early-onset use of tobacco or alcohol appeared to lead to an increase risk of using any illicit drugs, not limiting to ketamine.

### Implications

Our findings have implications for the application of ketamine expectancies in the prediction or intervention of ketamine use. First, avoiding any endorsement of positive ketamine expectancies is essential in preventing young people from the initiation of ketamine use. Previous studies have indicated that positive expectancies can predict future substance use. For example, young adolescents’ higher positive expectancies could predict subsequent adolescent problem drinking and greater drinking levels [70, 71] as well as adult alcohol use [29, 33].

Second, it is probably more challenging to enhance individuals’ awareness of negative ketamine expectancies because even ketamine users were on average aware of more than 3 negative expectancies. This implies that the inverse association of negative expectancies with ketamine use would not be detected unless the level of endorsed expectancies exceeded certain threshold (e.g., 5 negative expectancies). In other words, the relationship between negative expectancies and ketamine use is not linear. This might also explain previous findings that negative expectancies did not predict subsequent alcohol use [29, 33].

Third, modification of expectancies has been proposed as an intervention tool to reduce substance consumption [44]. People who were illicit drug-naïve might maintain or reinforce their endorsed negative expectancies and shun that of positive expectancies of ketamine use by learning from media or peer’s report on cognitive impairment or physical problems following such use [45]. As indicated in a meta-analysis of 62 studies of interventions for college drinking, the strategy to shape expectancies might be critical: a less confrontational feedback about alcohol expectancy was found to be more effective than those containing expectancy challenge [43]. Since the main expectations of ketamine use reported by our study participants were for entertainment (join in with others and make parties more fun), it warrants to devise a less confrontational feedback on decreasing an individual’s positive expectancies. Furthermore, it may also help to delay the involvement of exclusive ketamine users with other illicit drugs by elevating their endorsements of negative expectancies (e.g., make people less friendly). Empirical data revealed that ketamine users adopted some harm reduction strategies to minimize negative consequences of ketamine use, exemplifying the potential utility of raising awareness of ketamine-induced harms [19]. Given that the negative health consequences of ketamine use (e.g., urination problems) often emerge in relatively later temporal sequence than those of acute alcohol intoxication, expectancy modification should integrate peer network and personal experience while devising strategies, which is particularly true for young people.

### Limitations

This study has some limitations. First, the ketamine expectancy questionnaire used in this study was modified from that of marijuana use, not specifically designed for ketamine use. Since different substances might induce different anticipations and physical effects, each substance might need its own expectancy questionnaire to test the expectancy theory [45]. Therefore, our 12-item ketamine expectancy questionnaire might capture only the common effects for psychoactive substances, rather than fully represent the expectancies for ketamine use. Second, all data were self-reported. Although our data were collected using ACASI, the validity of information on sensitive issues, such as illicit drug use, might still be a question. Third, since this was a cross-sectional study, it did not allow us to infer causality on the relationships between ketamine expectancies and ketamine use. Lastly, as our results were derived from alcohol- and tobacco-using adults in Taipei metropolitan area, our findings may not be generalizable to other geographic areas of Taiwan or other populations, where the occurrence of ketamine usage might be different from ours.

## Conclusions

In conclusion, positive and negative expectancies exhibit differential relationships with different patterns of ketamine use. Positive and negative expectancies might mutually affect the decision and considering solely one dimension of expectancies might not predict the decision of ketamine use appropriately. Ketamine expectancies exhibit association with ketamine use independent of early-onset use of tobacco or alcohol. Our results indicate that a less confrontational feedback on decreasing an individual’s positive expectancies is essential in preventing young people from the initiation of ketamine use.

## Supplementary information

**Additional file 1: Table S1.** The proportion of lifetime ketamine use and male gender in each year of the RDS-sample in Taipei Metropolitan Area. **Table S2.** Univariate logistic regression analysis of any ketamine use on individual ketamine expectancies (unweighted) among 162 ever users of ketamine and 840 illicit drug-naive. **Table S3.** Multivariable logistic regression analysis of any ketamine use on ketamine expectancies (unweighted) among 162 ever users of ketamine and 840 illicit drug-naïve. **Table S4.** Ketamine-using history of the RDS-sample in Taipei Metropolitan Area (*N* = 1115), by illicit drug use experience**. Table S5.** Distribution of ketamine expectancies among early initiation of tobacco and alcohol, respectively. **Table S6.** Multinomial logistic regression model of illicit drug use experience (reference group: illicit drug-naïve) on continuous ketamine expectancies with adjustment for sociodemographics and early-onset tobacco use (*N* = 1115). **Table S7.** Multinomial logistic regression model of illicit drug use experience (reference group: illicit drug-naïve) on binary ketamine expectancies with adjustment for sociodemographics and early-onset alcohol use (*N* = 1112). **Table S8.** Multinomial logistic regression model of illicit drug use experience (reference group: illicit drug-naïve) on binary ketamine expectancies with interaction terms involving early-onset tobacco use (*N* = 1115). **Table S9.** Multinomial logistic regression model of illicit drug use experience (reference group: illicit drug-naïve) on binary ketamine expectancies with interaction terms involving early-onset alcohol use (*N* = 1112)

## Data Availability

The authors received permission to access the data used in this study; however, they are unable to share the data as they are not the data custodian.

## Abbreviations

ACASI: Audio computer-assisted self-interview
GHB: Gamma hydroxybutyrate
OR: Odds ratio
RDS: Respondent-driven sampling
