# Supplementary material for: Mis-anaesthetized society: expectancies and recreational use of ketamine in Taiwan
Source: BMC Public Health. 2019 Oct 17;19:1307. doi: 10.1186/s12889-019-7616-1 (PMC6798441; doi:10.1186/s12889-019-7616-1)
Supplement: Supplementary file 1 — Additional file 1: Table S1. The proportion of lifetime ketamine use and male gender in each year of the RDS-sample in Taipei Metropolitan Area. Table S2. Univariate logistic regression analysis of any ketamine use on individual ketamine expectancies (unweighted) among 162 ever users of ketamine and 840 illicit drug-naive. Table S3. Multivariable logistic regression analysis of any ketamine use on ketamine expectancies (unweighted) among 162 ever users of ketamine and 840 illicit drug-naïve. Table S4. Ketamine-using history of the RDS-sample in Taipei Metropolitan Area (N = 1115), by illicit drug use experience. Table S5. Distribution of ketamine expectancies among early initiation of tobacco and alcohol, respectively. Table S6. Multinomial logistic regression model of illicit drug use experience (reference group: illicit drug-naïve) on continuous ketamine expectancies with adjustment for sociodemographics and early-onset tobacco use (N = 1115). Table S7. Multinomial logistic regression model of illicit drug use experience (reference group: illicit drug-naïve) on binary ketamine expectancies with adjustment for sociodemographics and early-onset alcohol use (N = 1112). Table S8. Multinomial logistic regression model of illicit drug use experience (reference group: illicit drug-naïve) on binary ketamine expectancies with interaction terms involving early-onset tobacco use (N = 1115). Table S9. Multinomial logistic regression model of illicit drug use experience (reference group: illicit drug-naïve) on binary ketamine expectancies with interaction terms involving early-onset alcohol use (N = 1112) [file 12889_2019_7616_MOESM1_ESM.doc]

**Additional file**

**Table S1.** The proportion of lifetime ketamine use and male gender in each year of the RDS-sample in Taipei Metropolitan Area

**Table S2**. Univariate logistic regression analysis of any ketamine use on individual ketamine expectancies (unweighted) among 162 ever users of ketamine and 840 illicit drug-naive

**Table S3**. Multivariable logistic regression analysis of any ketamine use on ketamine expectancies (unweighted) among 162 ever users of ketamine and 840 illicit drug-naïve

**Table S4**. Ketamine-using history of the RDS-sample in Taipei Metropolitan Area (N = 1115), by illicit drug use experience

**Table S5**. Distribution of ketamine expectancies among early initiation of tobacco and alcohol, respectively

**Table S6**. Multinomial logistic regression model of illicit drug use experience (reference group: illicit drug-naïve) on continuous ketamine expectancies with adjustment for sociodemographics and early-onset tobacco use (N = 1115)

**Table S7.** Multinomial logistic regression model of illicit drug use experience (reference group: illicit drug-naïve) on binary ketamine expectancies with adjustment for sociodemographics and early-onset alcohol use (N = 1112)

**Table S8.** Multinomial logistic regression model of illicit drug use experience (reference group: illicit drug-naïve) on binary ketamine expectancies with interaction terms involving early-onset tobacco use (N = 1115)

**Table S9**. Multinomial logistic regression model of illicit drug use experience (reference group: illicit drug-naïve) on binary ketamine expectancies with interaction terms involving early-onset alcohol use (N = 1112)

Table S1. The proportion of lifetime ketamine use and male gender in each year of the RDS-sample in Taipei Metropolitan Area

|  | Total | Ketamine use | | |  | Male gender | | |
| --- | --- | --- | --- | --- | --- | --- | --- | --- |
| Year | N | n | %_wt_ | 95% CI |  | n | %_wt_ | 95% CI |
| 2007 | 144 | 14 | 6.5 | (5.5-50.0) |  | 82 | 52.9 | (50.0-58.5) |
| 2008 | 328 | 47 | 12.2 | (10.9-50.0) |  | 190 | 54.9 | (50.0-58.5) |
| 2009 | 350 | 57 | 15.1 | (12.4-50.0) |  | 258 | 69.9 | (50.0-72.6) |
| 2010 | 293 | 44 | 12.3 | (11.3-50.0) |  | 159 | 51.0 | (48.6-52.8) |
| Aggregate |  |  |  |  |  |  |  |  |
| 2007-2008 | 472 | 61 | 8.4 | (5.5-50.0) |  | 272 | 53.5 | (50.0-59.4) |
| 2009-2010 | 643 | 101 | 12.3 | (11.2-50.0) |  | 417 | 51.0 | (48.6-72.6) |
|  |  |  |  |  |  |  |  |  |
| 2007-2010 | 1115 | 162 | 11.8 | (9.4-15.0) |  | 689 | 57.2 | (51.5-62.9) |

Table S2. Univariate logistic regression analysis of any ketamine use on individual ketamine expectancies (unweighted) among 162 ever users of ketamine and 840 illicit drug-naive

| Ketamine expectancy item | OR | 95% CI |
| --- | --- | --- |
| Positive |  |  |
| P1 (stand up to others) | **2.69** | **(1.81-4.00)** |
| P2 (join in with others) | **2.34** | **(1.64-3.34)** |
| P3 (drive better) | 0.95 | (0.40-2.29) |
| P4 (make parties more fun) | **3.53** | **(2.47-5.05)** |
| P5 (enjoy a holiday) | **1.86** | **(1.30-2.66)** |
| P6 (make the world a better place) | 1.50 | (0.98-2.31) |
| Negative |  |  |
| N1 (lose controls and have accidents) | **0.38** | **(0.27-0.54)** |
| N2 (make people less friendly) | **0.30** | **(0.20-0.44)** |
| N3 (have a go at kids who are using) | 0.78 | (0.54-1.12) |
| N4 (don’t understand things when using) | **0.59** | **(0.42-0.83)** |
| N5 (break and destroy things when using) | **0.33** | **(0.24-0.46)** |
| N6 (have trouble remembering) | 0.90 | (0.61-1.33) |

Note: statistically significant results are highlighted in bold

Table S3. Multivariable logistic regression analysis of any ketamine use on ketamine expectancies (unweighted) among 162 ever users of ketamine and 840 illicit drug-naive

|  | Regression coefficient | | Area under |
| --- | --- | --- | --- |
| Model | aOR | 95% CI | ROC curve |
| 12-item model |  |  | 0.7800 |
| P1 (stand up to others) | **2.17** | **(1.3-3.62)** |  |
| P2 (join in with others) | **1.82** | **(1.08-3.05)** |  |
| P3 (drive better) | 0.88 | (0.33-2.33) |  |
| P4 (make parties more fun) | **3.47** | **(2.09-5.75)** |  |
| P5 (enjoy a holiday) | 0.97 | (0.6-1.59) |  |
| P6 (make the world a better place) | 0.65 | (0.37-1.15) |  |
| N1 (lose controls and have accidents) | **0.35** | **(0.21-0.58)** |  |
| N2 (make people less friendly) | **0.46** | **(0.29-0.73)** |  |
| N3 (have a go at kids who are using) | 1.43 | (0.81-2.52) |  |
| N4 (don’t understand things when using) | 1.02 | (0.6-1.71) |  |
| N5 (break and destroy things when using) | **0.28** | **(0.17-0.45)** |  |
| N6 (have trouble remembering) | 1.7 | (0.94-3.08) |  |
| 3-positive-item model (backward selection from positive expectancies; p-value threshold: 0.20) |  |  | 0.6744 |
| P1 (stand up to others) | **1.87** | **(1.22-2.87)** |  |
| P3 (drive better) | 0.52 | (0.21-1.31) |  |
| P4 (make parties more fun) | **3.14** | **(2.16-4.56)** |  |
| 8-item model (backward selection; p-value threshold: 0.20) |  |  | 0.7758 |
| P1 (stand up to others) | **2.14** | **(1.3-3.53)** |  |
| P2 (join in with others) | **1.86** | **(1.11-3.11)** |  |
| N6 (have trouble remembering) | **1.89** | **(1.08-3.31)** |  |
| P4 (make parties more fun) | **3.59** | **(2.18-5.91)** |  |
| N5 (break and destroy things when using) | **0.29** | **(0.18-0.46)** |  |
| P6 (make the world a better place) | 0.63 | (0.37-1.08) |  |
| N1 (lose controls and have accidents) | **0.38** | **(0.24-0.62)** |  |
| N2 (make people less friendly) | **0.47** | **(0.3-0.74)** |  |

Note: aOR = adjusted odds ratio; statistically significant results are highlighted in bold

Table S4. Ketamine-using history of the RDS-sample in Taipei Metropolitan Area (N = 1115), by illicit drug use experience

| Ketamine-using experience |  | Exclusive ketamine use  (N = 30) | | |  | Polydrug ketamine use  (N = 132) | | |  | Group comparisons |
| --- | --- | --- | --- | --- | --- | --- | --- | --- | --- | --- |
|  |  | N | %_wt_ | 95% CI |  | N | %_wt_ | 95% CI |  | P^a^ |
| Lifetime use ≥ 5 times |  | 8 | 24.3 | (8.6-47.0) |  | 82 | 61.5 | (47.9-74.2) |  | < .001 |
| Recent use ≤ 6 months |  | 10 | 23.1 | (7.7-45.0) |  | 49 | 33.2 | (20.8-47.6) |  | .287 |
| Frequency > 2 days/month |  | 4 | 6.3 | (0.6-15.3) |  | 28 | 19.7 | (10.1-31.8) |  | .088 |
|  |  | N | Mean | SD |  | N | Mean | SD |  | P^a^ |
| Age at first use |  | 30 | 18.7 | 3.5 |  | 132 | 20.5 | 4.9 |  | .058 |

^a^ χ^2^ test or Fisher’s exact test for categorical variables; ANOVA for quantitative variables

Table S5. Distribution of ketamine expectancies among early initiation, i.e., an onset age of <16 years, of tobacco and alcohol, respectively

|  | Tobacco use  (N = 1115) | | | | | | |  | Alcohol use  (N = 1112) | | | | | | |
| --- | --- | --- | --- | --- | --- | --- | --- | --- | --- | --- | --- | --- | --- | --- | --- |
|  | Early onset  (N = 406)  32%_wt_ (27.7-36.3) | |  | Late onset  (N = 709)  65.7%_wt_ (61.3-69.8) | | Group  Comparison | |  | Early onset  (N = 427)  34.1%_wt_ (30.1-38.6) | |  | Late onset  (N = 685)  65.7%_wt_ (61.3-69.8) | | Group  Comparison | |
| Ketamine expectancies | %_wt_ | 95% CI |  | %_wt_ | 95% CI |  | P^a^ |  | %_wt_ | 95% CI |  | %_wt_ | 95% CI |  | P^a^ |
| Positive sum, mean (SD) | 1.7 | (1.7) |  | 1.4 | (1.5) | <.001 | |  | 1.6 | (1.6) |  | 1.5 | 1.6 | .327 | |
| Negative sum, mean (SD) | 3.8 | (2.2) |  | 4.1 | (2.1) | .029 | |  | 3.9 | 2.2 |  | 4.1 | 2.2 | .081 | |
| Binary |  |  |  |  |  |  | |  |  |  |  |  |  |  | |
| High Positive | 60.6 | (53.7-67.4) |  | 57.9 | (52.1-64.3) | .392 | |  | 56.5 | (49.8-63.5) |  | 59.7 | (53.6-66.0) | .282 | |
| High Negative | 51.3 | (43.7-58.3) |  | 55.1 | (48.6-61.5) | .207 | |  | 45.8 | (38.4-53.0) |  | 57.9 | (51.3-64.4) | <.001 | |
| Combinations |  |  |  |  |  | .402 | |  |  |  |  |  |  | .002 | |
| Low Positive-High Negative | 13.1 | (8.9-18.1) |  | 15.4 | (11.9-19.7) |  | |  | 12.3 | (8.2-17.0) |  | 15.9 | (12.2-20.3) |  | |
| Low Positive-Low Negative | 26.7 | (20.0-32.5) |  | 27.1 | (21.1-34.2) |  | |  | 31.1 | (24.5-38.2) |  | 24.6 | (18.3-31.5) |  | |
| High Positive-High Negative | 38.6 | (31.7-45.7) |  | 39.6 | (33.5-45.4) |  | |  | 33.5 | (27-40.0) |  | 41.6 | (35.6-47.7) |  | |
| High Positive-Low Negative | 21.6 | (16.1-28.9) |  | 17.9 | (13.7-22.2) |  | |  | 23.1 | (17.2-29.3) |  | 17.8 | (13.5-22.4) |  | |

^a^ χ^2^ test or Fisher’s exact test for categorical variables; ANOVA for quantitative variables

Table S6. Multinomial logistic regression model of illicit drug use experience (reference group: illicit drug-naïve) on continuous ketamine expectancies with adjustment for sociodemographics and early-onset tobacco use (N = 1115)

|  | Any ketamine use | |  | The other illicit drug use | |
| --- | --- | --- | --- | --- | --- |
| Variables | aOR | 95% CI |  | aOR | 95% CI |
| *Model 1* |  |  |  |  |  |
| Male | 1.10 | (0.63-1.92) |  | 1.25 | (0.69-2.28) |
| Education < college | 1.48 | (0.83-2.63) |  | 0.79 | (0.44-1.44) |
| Unemployment | **3.91** | **(1.43-10.69)** |  | **4.74** | **(2.19-10.25)** |
| Age in years | **0.94** | **(0.91-0.97)** |  | 1.01 | (0.98-1.04) |
| Early-onset tobacco use | **1.96** | **(1.13-3.39)** |  | **2.26** | **(1.28-4.00)** |
| Positive ketamine expectancy | **1.62** | **(1.38-1.89)** |  | **1.26** | **(1.04-1.53)** |
| Negative ketamine expectancy | **0.77** | **(0.69-0.85)** |  | **0.82** | **(0.70-0.95)** |
| *Model 2* |  |  |  |  |  |
| Male | 1.17 | (0.65-2.11) |  | 1.28 | (0.71-2.29) |
| Education < college | 1.63 | (0.86-3.08) |  | 0.85 | (0.46-1.56) |
| Unemployment | **4.09** | **(1.57-10.67)** |  | **4.72** | **(2.26-9.82)** |
| Age in years | **0.94** | **(0.91-0.97)** |  | 1.01 | (0.98-1.04) |
| Early-onset tobacco use | **2.26** | **(1.27-4.03)** |  | **2.43** | **(1.4-4.2)** |
| Positive ketamine expectancy | **4.07** | **(2.17-7.6)** |  | **2.51** | **(1.32-4.78)** |
| Negative ketamine expectancy | 1.00 | (0.88-1.12) |  | 0.92 | (0.8-1.06) |
| Positive*Negative ketamine expectancy | **0.79** | **(0.71-0.89)** |  | **0.85** | **(0.75-0.96)** |

Note: statistically significant results are highlighted in bold

Table S7. Multinomial logistic regression model of illicit drug use experience (reference group: illicit drug-naïve) on binary ketamine expectancies with adjustment for sociodemographics and early-onset alcohol use (N = 1112)

|  | Any ketamine use | |  | The other illicit drug use | |
| --- | --- | --- | --- | --- | --- |
| Variables | aOR | 95% CI |  | aOR | 95% CI |
| *Model 1* |  |  |  |  |  |
| Male | 1.18 | (0.67-2.08) |  | 1.27 | (0.71-2.27) |
| Education < college | 1.66 | (0.94-2.93) |  | 0.97 | (0.54-1.73) |
| Unemployment | **4.90** | **(1.69-14.16)** |  | **5.29** | **(2.51-11.15)** |
| Age in years | **0.95** | **(0.91-0.98)** |  | 1.02 | (0.99-1.05) |
| Early-onset alcohol use | 1.58 | (0.90-2.76) |  | **2.90** | **(1.62-5.17)** |
| High Positive expectancies | **10.59** | **(5.54-20.26)** |  | **3.19** | **(1.61-6.32)** |
| High Negative expectancies | **0.15** | **(0.08-0.26)** |  | **0.43** | **(0.22-0.84)** |
| *Model 2* |  |  |  |  |  |
| Male | 1.18 | (0.67-2.09) |  | 1.26 | (0.71-2.26) |
| Education < college | 1.66 | (0.93-2.96) |  | 0.97 | (0.54-1.74) |
| Unemployment | **4.88** | **(1.73-13.8)** |  | **5.14** | **(2.50-10.59)** |
| Age in years | **0.95** | **(0.91-0.98)** |  | 1.02 | (0.99-1.05) |
| Early-onset alcohol use | 1.63 | (0.92-2.87) |  | **3.01** | **(1.69-5.35)** |
| Ketamine expectancy combination  (ref: Low Positive-High Negative) |  |  |  |  |  |
| Low Positive-Low Negative | **4.76** | **(1.30-17.37)** |  | 0.78 | (0.33-1.83) |
| High Positive-High Negative | **7.57** | **(2.11-27.16)** |  | 1.23 | (0.57-2.65) |
| High Positive-Low Negative | **57.89** | **(16.68-200.95)** |  | **4.61** | **(1.89-11.21)** |

Note: (1) high or low expectancies are divided by the median of the illicit drug-naïve

(2): statistically significant results are highlighted in bold

Table S8. Multinomial logistic regression model of illicit drug use experience (reference group: illicit drug-naïve) on binary ketamine expectancies with interaction terms involving early-onset tobacco use (N = 1115)

|  | Any ketamine use | |  | The other illicit drug use | |
| --- | --- | --- | --- | --- | --- |
| Variables | aOR | 95% CI |  | aOR | 95% CI |
| *Model 1* |  |  |  |  |  |
| Male | 1.21 | (0.68-2.14) |  | 1.28 | (0.7-2.35) |
| Education < college | 1.48 | (0.84-2.61) |  | 0.83 | (0.46-1.49) |
| Unemployment | **4.77** | **(1.59-14.37)** |  | **5.45** | **(2.54-11.67)** |
| Age in years | **0.94** | **(0.91-0.98)** |  | 1.01 | (0.98-1.04) |
| Early-onset tobacco use (EOT) | 2.16 | (0.77-6.03) |  | 2.43 | (0.93-6.32) |
| High Positive expectancies (HPE) | **9.38** | **(3.98-22.12)** |  | 2.59 | (0.93-7.21) |
| High Negative expectancies (HNE) | **0.14** | **(0.06-0.32)** |  | 0.51 | (0.19-1.35) |
| EOT * HPE | 1.2 | (0.34-4.24) |  | 1.52 | (0.37-6.21) |
| EOT * HNE | 1.0 | (0.29-3.5) |  | 0.56 | (0.15-2.14) |
| *Model 2* |  |  |  |  |  |
| Male | 1.21 | (0.68-2.15) |  | 1.26 | (0.69-2.31) |
| Education < college | 1.47 | (0.82-2.62) |  | 0.82 | (0.45-1.48) |
| Unemployment | **4.7** | **(1.6-13.77)** |  | **5.29** | **(2.52-11.09)** |
| Age in years | **0.95** | **(0.91-0.98)** |  | 1.01 | (0.98-1.04) |
| Early-onset tobacco use (EOT) | 5.29 | (0.85-33) |  | 1.1 | (0.25-4.79) |
| Ketamine expectancy combination  (ref: Low Positive-High Negative) |  |  |  |  |  |
| Low Positive-Low Negative (C1) | **6.89** | **(1.62-29.23)** |  | 0.52 | (0.17-1.64) |
| High Positive-High Negative (C2) | **9.26** | **(2.18-39.31)** |  | 0.93 | (0.36-2.41) |
| High Positive-Low Negative (C3) | **69.77** | **(19.03-255.79)** |  | 3.3 | (0.98-11.14) |
| EOT * C1 | 0.36 | (0.04-3.03) |  | 3.23 | (0.5-20.62) |
| EOT * C2 | 0.45 | (0.06-3.53) |  | 2.18 | (0.4-12) |
| EOT * C3 | 0.53 | (0.07-4.07) |  | 2.85 | (0.42-19.17) |

Note: (1) high or low expectancies are divided by the median of the illicit drug-naïve

(2): statistically significant results are highlighted in bold

Table S9. Multinomial logistic regression model of illicit drug use experience (reference group: illicit drug-naïve) on binary ketamine expectancies with interaction terms involving early-onset alcohol use (N = 1112)

|  | Any ketamine use | |  | The other illicit drug use | |
| --- | --- | --- | --- | --- | --- |
| Variables | aOR | 95% CI |  | aOR | 95% CI |
| *Model 1* |  |  |  |  |  |
| Male | 1.17 | (0.66-2.05) |  | 1.25 | (0.7-2.25) |
| Education < college | 1.69 | (0.95-3.01) |  | 0.99 | (0.55-1.77) |
| Unemployment | **5.15** | **(1.79-14.81)** |  | **5.58** | **(2.61-11.91)** |
| Age in years | **0.94** | **(0.91-0.98)** |  | 1.02 | (0.99-1.05) |
| Early-onset alcohol use (EOA) | **3.49** | **(1.15-10.58)** |  | **3.66** | **(1.41-9.46)** |
| High Positive expectancies (HPE) | **18.92** | **(6.99-51.19)** |  | **4.54** | **(1.57-13.17)** |
| High Negative expectancies (HNE) | **0.13** | **(0.06-0.28)** |  | **0.34** | **(0.13-0.89)** |
| EOA * HPE | 0.32 | (0.08-1.2) |  | 0.49 | (0.12-2) |
| EOA * HNE | 1.29 | (0.4-4.12) |  | 1.62 | (0.44-5.97) |
| *Model 2* |  |  |  |  |  |
| Male | 1.17 | (0.66-2.06) |  | 1.25 | (0.7-2.24) |
| Education < college | 1.7 | (0.95-3.04) |  | 1 | (0.55-1.79) |
| Unemployment | **5.13** | **(1.83-14.44)** |  | **5.46** | **(2.63-11.35)** |
| Age in years | **0.95** | **(0.91-0.98)** |  | 1.02 | (0.99-1.05) |
| Early-onset alcohol use (EOA) | 5.22 | (0.61-44.44) |  | **5.98** | **(1.67-21.49)** |
| Ketamine expectancy combination  (ref: Low Positive-High Negative) |  |  |  |  |  |
| Low Positive-Low Negative (C1) | **5.34** | **(1.14-24.88)** |  | 0.98 | (0.28-3.44) |
| High Positive-High Negative (C2) | **13.94** | **(3.39-57.28)** |  | 1.85 | (0.66-5.16) |
| High Positive-Low Negative (C3) | **110.64** | **(29.46-415.52)** |  | **7.7** | **(2.13-27.75)** |
| EOA * C1 | 0.65 | (0.06-7.47) |  | 0.63 | (0.11-3.55) |
| EOA * C2 | 0.26 | (0.03-2.69) |  | 0.45 | (0.1-2.09) |
| EOA * C3 | 0.23 | (0.02-2.35) |  | 0.35 | (0.06-2.01) |

Note: (1) high or low expectancies are divided by the median of the illicit drug-naïve

(2): statistically significant results are highlighted in bold
